# The association between indwelling urinary catheter use in the elderly and urinary tract infection in acute care

**DOI:** 10.1186/1471-2318-6-15

**Published:** 2006-10-12

**Authors:** Susan E Hazelett, Margaret Tsai, Michele Gareri, Kyle Allen

**Affiliations:** 1 41 Arch St., room 212, Summa Health System, Akron, Ohio, 44304, USA

## Abstract

**Background:**

The use of indwelling urinary catheters (IUCs) is thought to be the most significant risk factor for developing nosocomial urinary tract infections (UTIs). However, it is unclear how many elderly patients have preexisting bacteriuria prior to IUC placement. The purpose of this study was to determine 1) the frequency and appropriateness of IUC use in the Emergency Department (ED) in elderly patients admitted to our acute care hospital, 2) the percentage of elderly patients with an IUC who were discharged from the hospital with a diagnosis of UTI, 3) the percentage of patients with IUCs who were diagnosed and treated for UTI in the ED or who had admission bacteriuria ≥10^5 ^organisms/ml indicating preexisting UTI, and 4) the percentage of patients with no indication of UTI on admission who had inappropriately placed IUCs and subsequently were diagnosed with a UTI.

**Methods:**

Retrospective chart review. Chi square used to test significance of differences in proportions.

**Results:**

Seventy three percent of patients who received an IUC in the ED were elderly (≥65 years old). During the study period, 277 elderly patients received an IUC prior to admission. Of these, 77 (28%) were diagnosed with UTI during their hospitalization. Fifty three (69%) of those diagnosed with a UTI by discharge either had the UTI diagnosed in the ED or had bacteriuria ≥10^5 ^organisms/ml prior to IUC placement. Of the 24 elderly patients who developed a catheter-associated UTI (i.e., 9% of the elderly population who received an IUC), 11 of the IUCs were placed inappropriately. Thus, 4% of elderly patients with no indication of UTI on admission who received an inappropriate IUC in the ED had a primary or secondary diagnosis of UTI by discharge. The overall rate of nosocomial UTI due to an inappropriately placed IUC was the same in males and females.

**Conclusion:**

This study indicates that the strong association between IUC use and UTI may be partly explained by the high prevalence of preexisting UTI prior to IUC placement. Further prospective studies are needed to clarify the true risk vs benefit ratio for IUC use in acutely ill elderly patients.

## Background

Approximately 4 million people each year receive an indwelling urinary catheter (IUC) [1] and 5–20% of hospitalized patients who receive an IUC will be diagnosed with a urinary tract infection (UTI) [1,2]. IUC use is thought to be the most significant risk factor for developing nosocomial UTIs, especially in acutely ill elderly patients [3-6]. Indeed, the leading category of nosocomial infections are UTIs [7], and 80% of these are associated with IUCs [1]. UTIs are associated with increased morbidity, mortality, length of stay, and costs [4,8-11]. Even with improvements in nursing care of the catherized patient and experimental redesign of catheters themselves, UTI remains a problem in the catherized patient [1,10,12-15]. It is important, therefore, to carefully weigh the risks vs benefits in patients for whom an IUC is being considered and to minimize the inappropriate use of IUCs.

The association between UTIs and IUCs in acutely ill patients is well documented. However, this association does not establish cause and effect and most studies that establish the association between IUC and UTI do not examine whether the UTI preceeded IUC placement. One study by Gardam et al [5] showed that 2 of 24 (8.3%) consecutive patients with IUCs in the Emergency Department (ED) had a UTI prior to catheter insertion, and 5 of the 24 (20.8%) developed a catheter-associated UTI during their hospital stay. Three of these 5 UTIs were associated with inappropriate IUC placement. However, this study was small, urine cultures were not obtained consistently, and it included patients of all ages.

The presence of a preexisting UTI may be more likely in elderly patients admitted to the hospital [1,8]. Previous studies have found that asymptomatic bacteriuria affects up to 50% of noninstitutionalized geriatric women and 30% of geriatric men [9]. Johansson et al [11] found that 38% of elderly patients with hip fractures had positive urine cultures on admission to the hospital. In addition, O'Donnell et al [16] point out that 20–50% of nursing home residents have asymptomatic bacteriuria, and this rises to 100% in patients with IUCs. Several factors predispose the elderly to UTIs including functional abnormalities (e.g., enlarged prostate gland, obstructions), chronic diseases (e.g., diabetes, cerebrovascular disease, and neurodegenerative diseases such as Parkinson's and multiple sclerosis), and certain medications.

The current study attempts to replicate the findings of Gardam et al [5] with a larger sample. In addition, this study focuses exclusively on elderly patients (i.e., those ≥65 years old).

The purpose of this study was to 1) examine the frequency and appropriateness of IUC use in the ED in elderly patients admitted to our acute care hospital, 2) to determine the percentage of elderly patients with an IUC who were discharged from the hospital with a primary or secondary diagnosis of UTI, 3) to determine the percentage of patients with IUCs who were diagnosed and treated for UTI in the ED or who had admission bacteriuria ≥10^5 ^organisms/ml indicative of preexisting UTI, and 4) to determine the percentage of elderly patients with no indication of UTI on admission who had inappropriately placed IUCs and subsequently were diagnosed with a UTI.

## Methods

This study used retrospective chart review of all patients admitted to our acute care hospital from the ED with an IUC from March 1–31, 2004. Per hospital policy, all catheters were placed using sterile technique. Data collected included the patient's age, diagnoses, results of urine cultures, and the reason for IUC use. The significance of differences in proportions was determined using Chi square. Permission to conduct this study was obtained from our institution's Institutional Review Board (IRB).

For the purposes of this study, the presence of a UTI on admission was defined as 1) an admission urine culture with ≥10^5 ^organisms/ml or 2) the diagnosis and treatment of UTI by the ED physician. Urine cultures positive only for urogenital flora were considered negative for UTI for this study.

Catheter appropriateness was determined using the criteria of Nickel [3]. IUCs were considered appropriate for surgery, accurate measurement of intake and output, urinary retention, urinary incontinence posing a risk to the patient, urinary obstruction, altered blood pressure or blood volume status requiring accurate urine measurement, urine measurement in an uncooperative patient, bladder irrigation for a urinary tract hemorrhage, and palliative care for the terminally ill. This information was obtained by chart review by Registered Nurses.

## Results

### Reason for IUC placement in the elderly

**Figure 1:**
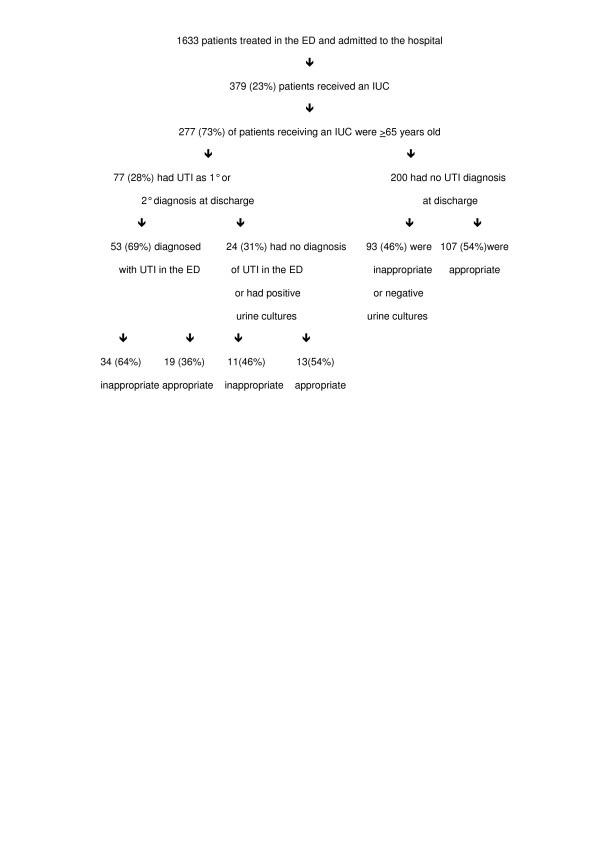
Association between IUC use and UTI in an acutely ill elderly population.

Between March 1^st ^and March 31^st ^2004, 1633 patients were admitted to our acute care hospital from the ED. Of these patients, a total of 379 patients received an IUC (figure 1). Of these, 277 (73%) were patients ≥65 years old. Significantly more elderly patients received and IUC than those < 65 years old (30%vs 12%, respectively, p < .0001). Overall, 139 (51%) of the elderly had IUCs appropriately placed and 138 (49%) had inappropriately placed IUCs. Table 1 shows the primary reason patients received an IUC as determined by chart review. It also shows the number of patients with an IUC for each reason who had a UTI at admission, who had a UTI diagnosed by discharge, and the number who developed a nosocomial UTI.

**Table 1:** Primary reason for indwelling urinary catheter placement

**Clinical Indication**	**Number of patients with IUC for this reason**	**Number with IUC for this reason who had UTI diagnosis by discharge**	**Number with IUC for this reason who had UTI diagnosis at admission**	**Nosocomial UTI**
Confusion	42	21	18	3
Fractured Hip	17	1	0	1
Intubated/IntensiveCareUnit/CriticalCareUnit admission	36	6	4	3
Pneumonia	4	0	0	0
Rectal bleed	1	0	0	0
Intake&Output	7	5	3	2
Weakness	4	2	2	0
Surgical case	21	2	1	1
Dehydration	6	3	2	1
Unresponsive/Loss of conciousness	14	4	2	0
Short of breath	4	1	0	1
Unable to ambulate	9	4	2	1
Do Not Resucitate status	2	0	0	0
Preexisting catheter	3	3	2	1
Restrained	2	1	1	0
Renal failure	2	1	0	1
Urinary retention	3	1	1	0
IntensiveCareUnit admission looked likely at admission	4	0	0	0
Trauma	4	0	0	0
Blood in urine	3	0	1	0
Fall/syncope prior to arrival	11	3	2	3
stroke/hemiplegia	7	1	2	0
Abdominal pain	7	4	3	1
Urinalysis/cultures	13	5	4	1
CongestiveHeartFailure	42	7	1	4
Cardiac catherization	1	0	0	0
Gastrointestinal bleed	2	0	0	0
Incontinent prior to arrival	3	2	2	0
Cellulitis	1	0	0	0

### Percentage of catherized patients who developed a nosocomial UTI

Seventy seven (28%) of the 277 elderly patients who received an IUC had UTI as a primary or secondary diagnosis at discharge. These patients ranged in age from 65 to 101 years old and 32 (42%) were from a nursing home. Only 57 of the 77 (74%) had urine cultures performed, 40 of which were positive and 17 were negative. Four of the 77 were being treated for a UTI prior to arrival in the ED, 3 had urosepsis, 34 displayed clinical signs/symptoms of UTI, and 36 had asymptomatic UTIs. Significantly more patients with an IUC had a UTI diagnosed by discharge than those with no IUC (28% vs 10%, respectively, p < .0001). Of the 77, 53 (69%) were diagnosed and treated for UTI in the ED or had urine cultures showing ≥10^5 ^organisms/ml, leaving 24 elderly patients without a UTI on admission who received an IUC and subsequently developed a UTI. In other words, 9% of the elderly population who received an IUC developed a nosocomial UTI.

### Association between nosocomial UTIs and inappropriately placed IUCs

Of the 24 elderly patients who developed a nosocomial UTI, 11 of the IUCs were determined to have been placed inappropriately (Table 2). Thus, 46% of the nosocomial UTIs were due to inappropriately placed IUCs. Overall 11 of the 277 (4%) patients ≥65 years old who received an IUC developed a nosocomial UTI associated with an inappropriately placed IUC.

**Table 2:** Reason for indwelling urinary catheters in patients who developed a nosocomial urinary tract infection by appropriateness (n = 24)

**Appropriate (n = 13)**	
**Diagnosis**	**Number**

Congestive Heart Failure	4
Renal failure	1
Intake and output	2
Intensive Care Unit admission	3
Hip fracture	1
Surgical candidate	1
Urology patient (catheter prior to arrival)	1
**Inappropriate (n = 11)**	
Urinalysis/cultures	1
Dehydration	1
Fall/syncope	3
Abdominal pain	1
Decreased mental status	3
Short of breath	1
Unable to ambulate	1

Of the 200 elderly patients who received an IUC but were not diagnosed with a UTI during their hospital stay, 93 (46%) of the IUCs were placed inappropriately, while 107 (54%) were appropriately placed.

### Gender differences

**Figure 2:**
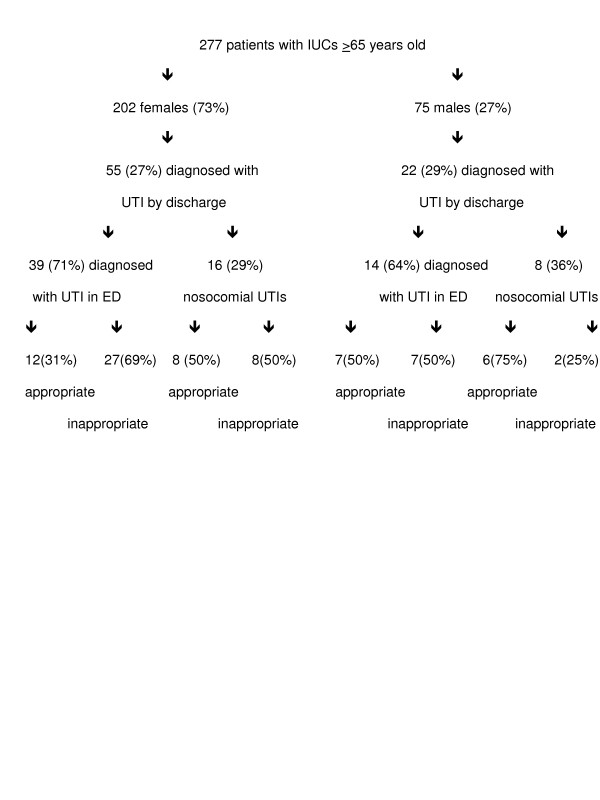
Gender differences in IUC outcomes.

A significantly greater proportion of elderly patients receiving an IUC were female vs male (73% vs 27% respectively, p < .0001) (figure 2). Both groups, however, had similar rates of UTI as a primary or secondary diagnosis at discharge (27% females, 29% males). Of those diagnosed with UTI by discharge, both groups also had similar rates of nosocomial UTI (29% females, 36% males). In females with nosocomial UTIs, IUC placement was inappropriate 50% of the time while in males with nosocomial UTIs, IUC placement was inappropriate only 25% of the time. Overall, 4% of all elderly females and 3% of all elderly males developed a nosocomial UTI after having an IUC placed inappropriately.

## Discussion

IUCs are routinely cited as the primary cause of nosocomial infections [3-6]. On the other hand, previous studies have shown that decreasing IUC use is not associated with a decrease in UTIs [17]. The present study showed that 28% of elderly patients who received an IUC were diagnosed with a UTI during their hospitalization. These data support the strong association between IUC use and a diagnosis of UTI that has been shown in previous studies. However, closer examination of the clinical data reveals that 53 (69%) of the patients who were diagnosed with UTI had either clinical signs/symptoms of UTI that were diagnosed and treated in the ED or they had an admission bacteriuria ≥10^5 ^organisms/ml indicative of a preexisting UTI. Thus, only 9%, rather than 28%, of elderly patients who received an IUC developed a nosocomial UTI. Thus, this study suggests that when preexisting UTIs are eliminated from the data, the association between IUC use and nosocomial UTI may be weaker than has been previously thought.

There is general agreement that the use of IUCs is appropriate in specific clinical situations. None-the-less, previous studies have shown that when using agreed upon definitions of appropriateness for IUC use, only about 50% of IUCs are used appropriately [2,18]. Our results support these previous findings by showing that in this elderly population with IUCs who developed a nosocomial UTI, only 54% of the IUCs were placed appropriately. However, this means that only 11 of the 277 (4%) patients ≥65 years old who received an IUC developed a nosocomial UTI associated with an inappropriately placed IUC. Thus, when IUC appropriateness is taken into consideration, the proportion of preventable nosocomial UTIs may also be smaller than previously thought.

In this study, female elderly patients were more susceptible to inappropriate IUC use than their male counterparts. Indeed, only two (25%) male patients who developed a nosocomial UTI had an IUC placed inappropriately, while 8 (50%) females who developed a nosocomial UTI had an inappropriately placed IUC. However, overall males and females were essentially equal in their rate of nosocomial UTI associated with an inappropriately placed IUC.

The main limitation of this study was the use of retrospective chart review (with its inherent problems of missing data) to determine the appropriateness of IUC placement. As has been noted in previous studies, the reason for IUC placement was rarely explicitly stated in the chart and therefore had to be deduced from the available clinical information. However, the lack of documentation regarding the reason for IUC placement would only result in an underestimation of IUCs that were appropriate using the criteria established by Nickel [3]. For example, Nickel's criteria for appropriateness allows for catheter use for urine measurement in uncooperative patients. In our sample, the largest number of inappropriate IUCs was for confused patients, some of whom were undoubtedly uncooperative and therefore may have actually had an appropriately placed IUC. To ensure the most reliable interpretation of the reason for IUC placement from the chart review, Registered Nurses, one of whom was an ED nurse, were used as chart reviewers.

Rarely did it appear that there was a single reason for IUC placement. Indeed, it seemed in most cases that there were several patient characteristics contributing to the decision to use an IUC. For example, some patients were confused, dehydrated, and syncopal prior to arrival in the ED. Some had significant drops in their oxygen saturation levels when they were moved around in the bed. Some were in pain, immobile, and incontinent. While any one of these reasons alone may not meet the criteria for an appropriately placed IUC, we should consider the possibility that, taken together, the combination of clinical factors may tip the risk benefit scale. Perhaps appropriateness needs to be redefined on an individual basis when there is a constellation of factors that may contribute to IUC use.

Another limitation of this study is the fact that we did not record the duration of IUC use in relation to nosocomial UTI development. Numerous studies demonstrate that the risk of UTI increases as the duration of catherization increases [9,12,18-20]. Unfortunately, in this retrospective study we were unable to look at this relationship due to inconsistent documentation by nursing staff regarding the timing of IUC discontinuation.

The use of antibiotics might confound the results seen here. We did not examine correlations between antibiotic use and UTI in patients with IUCs due to the unreliable nature of pre-hospitalization medication histories. We know, however, that some patients who were diagnosed with UTI were being treated with antibiotics for various reasons prior to ED admission, some were treated with antibiotics in the ED, and some were treated with antibiotics on the nursing unit. Furthermore, we have no evidence to suggest that antibiotic use in our sample was substantially different from that which would be seen in any typical ED population. Thus, the effects of antibiotic use on the generalizability of our findings may be minimal.

A final limitation of this study may be the criteria used for a positive UTI (i.e., diagnosis and treatment of UTI by the ED physician or ≥10^5^organisms/ml in admission urine culture). While some authors define UTI simply by colony counts in urine cultures [21], others say that a positive diagnosis depends on the presence of UTI signs/symptoms and a positive urine culture [9]. Some authors define UTI as ≥10^3 ^organisms/ml [23] and state that >90% of these patients are asymptomatic. As stated by Nicolle [22], "neither a positive urine culture nor clinical presentation allows a diagnosis of symptomatic urinary infection to be made with a high degree of certainty" in the elderly. As with other studies, our definition is not optimal since UTI may be over-diagnosed and treated by ED physicians. Unfortunately, urine cultures are not routinely obtained when UTI is diagnosed and treated in the ED, making verification of the diagnosis difficult. However, there is also evidence from this study that UTI may be under-diagnosed in the ED as 18 patients had positive cultures but were not diagnosed with UTI in the ED. We chose ≥10^5 ^organisms/ml or the presence of UTI symptoms prompting treatment in the ED as indicators of UTI which is in line with the Center for Disease Control and other national guidelines defining UTI [24,25].

## Conclusion

The results of this study indicate that IUC use following an emergency admission may not pose as high a risk for nosocomial UTI as previously thought, nor are they as highly associated with preventable UTIs. These findings run contrary to conventional wisdom and to our own expectations. Further research using better documentation for the reason for IUC use and the duration of use is warranted. Furthermore, despite these findings there is still a sizeable percentage of patients who develop nosocomial UTIs after the inappropriate placement of an IUC. Given the morbidity and mortality associated with UTIs in acutely ill elderly patients, it is still reasonable to make every effort to limit the use of IUCs to only those patients for whom an IUC is appropriate. Finally, the indiscriminant use of IUCs in acute care hospitals should still be avoided in the elderly due to the associated increased risk of functional impairment, falls, and immobility. Further research is needed to clarify the relationship between IUC use and preventable UTIs so that clinicians can more accurately assess the risks compared to the benefits of IUC use.

## Competing interests

The author(s) declare that they have no competing interests.

## Authors' contributions

SH contributed to the study design, data collection, data analysis, and manuscript preparation. MT contributed to the study design, data collection, and manuscript preparation. MG contributed to study design, data collection, and manuscript preparation. KA conceived the study and contributed to the study design and manuscript preparation. All authors read and approved the final manuscript.

## Pre-publication history

The pre-publication history for this paper can be accessed here:


